# Artificial intelligence (AI)-powered bibliometric analysis of global trends in mesenchymal stem cells (MSCs)-derived exosome research: 2014–2023

**DOI:** 10.37796/2211-8039.1470

**Published:** 2024-12-01

**Authors:** Shih-Chang Tsai, Bing-Han Wan, Fuu-Jen Tsai, Jai-Sing Yang

**Affiliations:** a Department of Biological Science and Technology, China Medical University, Taichung, Taiwan; b School of Chinese Medicine, College of Chinese Medicine, China Medical University, Taichung, Taiwan; c China Medical University Children’s Hospital, Taichung, Taiwan; d Department of Medical Genetics, China Medical University Hospital, Taichung, Taiwan; e Department of Medical Research, China Medical University Hospital, China Medical University, Taichung, Taiwan

**Keywords:** Exosomes, Mesenchymal stem cells (MSCs), Web of Science, Artificial intelligence (AI)-bibliometric tools, CiteSpace, VOSviewer

## Abstract

**Introduction:**

In recent years, significant progress has been made in regenerative medicine, specifically in using mesenchymal stem cells (MSCs) due to their regenerative and differentiating abilities. An exciting development in this area is the utilization of exosomes derived from MSCs, which have shown promise in tissue restoration, immune system modulation, and cancer treatment.

**Objectives:**

This study aims to analyze global research trends and the academic impact of MSCs-derived exosomes from 2014 to 2023, providing a comprehensive overview of this emerging field.

**Materials and methods:**

The Web of Science database selected 948 relevant publications from 2014 to 2023. Artificial intelligence (AI)-bibliometric tools, including Bibliometrix, CiteSpace, and VOSviewer, were employed to analyze and visualize the data. The focus was on publication quantity, research nations, institutional partnerships, keywords, and research focal points.

**Results:**

The study revealed that China, Japan, Taiwan, and the United States are the leaders in publication volume and impact in MSCs-derived exosome research. China has the highest number of publications, while the United States and Iran excel in research quality and influence. Primary research themes were identified through keyword and clustering analyses, including tissue repair, immune modulation, bone regeneration, and cancer treatment. The study also emphasized the importance of international collaboration, with China and the United States demonstrating the most robust cooperation.

**Conclusion:**

MSCs-derived exosome research rapidly expands worldwide, showing promising prospects in regenerative medicine and cell therapy. With continued research and international collaboration, MSCs-derived exosomes are expected to play a vital role in future therapeutic application.

## 1. Introduction

Mesenchymal stem cells (MSCs), first discovered in the 1970s and later given their name in the 1980s, have been the subject of numerous studies [1–5]. This is because they can self-renew and change into different types of cells, like osteocytes, adipocytes, and chondrocytes. MSCs exhibit strong anti-inflammatory and immune-modulatory characteristics, making them valuable candidates in regenerative medicine [6–8]. More than 950 clinical trials have explored their therapeutic potential, particularly for osteoarthritis, cardiovascular diseases, and neurological disorders [9,10]. Because these qualities and MSCs can speed up tissue regeneration through paracrine effects and immune modulation, they are even more critical in tissue engineering and regenerative medicine [11,12].

Although significant progress has been made, there are still knowledge gaps regarding the complete range of MSCs paracrine functions, especially concerning exosomes. These secreted nano-vesicles contain proteins, lipids, and genetic material and play a crucial role in intercellular communication and immune modulation [13,14]. MSCs-derived exosomes have a lot of potential in areas like cancer treatment and tissue regeneration, mainly because they can affect immune responses and help tissues heal. However, according to Marote et al. and Yin et al. [15,16], researchers still don’t fully understand how they function, where they go in the body, or how cells absorb them. Exosomes, tiny extracellular vesicles ranging from 30 to 120 nm in size and released by cells, have attracted considerable attention for their involvement in cell-to-cell communication, immune system regulation, and the progression of various diseases [17–19].

Exosomes made from MSCs have shown promise in stopping cancer from spreading and worsening by changing immune responses and the environment around the tumor [15,20]. Exosome-based therapies are better than traditional cellular therapies in regenerative medicine because they are safer, there is less chance of immune rejection, and tumors are less likely to form [21]. With nations such as China, the United States, Japan, and Germany investing in MSCs-derived exosome research, there is an increasing demand to identify global patterns, partnerships, and primary research areas in this burgeoning field [22–24]. However, a complete artificial intelligence (AI)-bibliometric study of MSCs-derived exosome research has not yet been done.

This makes understanding the research landscape and spotting critical collaborative networks harder. Such an analysis could illuminate the leading research institutions and countries, as well as the crucial themes shaping the future of this domain [25]. This research addresses existing knowledge gaps by conducting a thorough AI-bibliometric analysis of MSCs-derived exosome research from 2014 to 2023, leveraging resources like the Web of Science. Employing AI-bibliometric software such as Bibliometrix, CiteSpace, and VOSviewer, the study will examine publication output, institutional collaborations, research focus areas, and citation patterns. The main objective is to find crucial global research themes, collaborative frameworks, and research trends in MSCs-derived exosome studies, focusing on how they can be used in cell therapy and regenerative medicine.

Using heat maps and network diagrams to show data will help scientists, decision-makers, and medical professionals understand it better. This will encourage international cooperation and make it easier to use MSCs-derived exosomes in the clinic [18,21,26]. Ultimately, this study will fill existing knowledge gaps, identify new trends, and provide a framework for advancing therapies using exosomes derived from MSCs. By employing artificial intelligence (AI)-bibliometric analysis, the evolution of this field will be thoroughly documented, offering valuable insights to newcomers and helping to direct resources toward the most promising research areas.

## 2. Methods

### 2.1. Data source and search strategy

This study employs AI-bibliometric analysis, drawing data from the Science Citation Index Expanded (SCI-E) edition of the Web of Science Core Collection (WoSCC) database. The China Medical University Library accessed this database on April 26, 2024. This study focuses on English-language publications from 2014 to 2023 to ensure a thorough and credible analysis. This period reflects the significant advancements in exosome research. The search strategy involved querying titles using the following specific terms: TI = ((mesenchymal stem cells) or (mesenchymal stem cell)) and TI = exosomes and TI = derived. The primary strategy for identifying pertinent literature employed this methodology. Initially, 1194 documents were identified in the search. After filtering for only “Article” and “Review Article” document types, the final dataset was narrowed to 948 relevant publications. This meticulously selected collection of documents is the foundation for a comprehensive analysis of research trends in MSCs-derived exosomes.

### 2.2. Data analysis and visualization by artificial intelligence (AI)

**Fig. 1:**
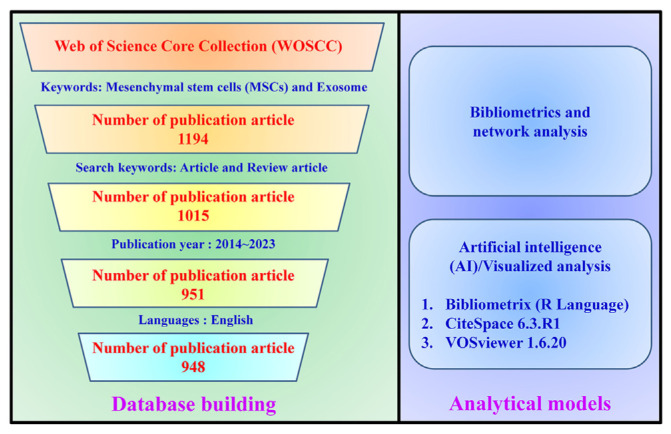
AI-Bibliometrics and network analysis of MSCs and exosome research using AI and visualization tools. The diagram presents a systematic process for constructing a publications database and leveraging visual analytics to examine research patterns in the MSCs and exosome fields.

After organizing and screening the literature, this study employed AI-bibliometric analysis and data visualization tools [27]. Specifically, the research utilized VOSviewer, Bibliometrix, and CiteSpace to conduct visual analyses and generate maps and clusters (see Fig. 1 for a flowchart illustrating the literature search and screening process). VOSviewer is a specialized tool for creating visual representations of bibliometric networks and grouping-related elements. It uses various colors to differentiate clusters, allowing researchers to identify patterns and connections in large bibliographic datasets. The study used VOSviewer software (version 1.6.20), which visualizes bibliometric networks, such as co-citation networks of articles, journals, countries, institutions, networks of authors working together, and maps showing how keywords appear together. Similarly, colored nodes serve as cluster representations, with larger nodes denoting higher publication counts. The thickness of the links indicates the connection strength between nodes. The overall collaboration level was quantitatively assessed by calculating the total link strength (TLS). VOSviewer was selected because of its intuitive interface and capability to cluster information related to countries, institutions, journals, authors, citations, and keywords [28]. Bibliometrix, an R-based software package, examined and graphically represented social network diagrams. This software facilitated the investigation of prominent journals, countries, and institutions, and the creation of word clouds derived from authors’ keywords. Additionally, it helped to assess the contributions and collaborative efforts of various countries. The advanced bibliometric software CiteSpace was employed to analyze the networks of citations, keywords, and author collaborations. This software provides valuable insights into research trends, key focus areas, and emerging frontiers in a particular field. The study utilized CiteSpace version 6.3.R1 for keyword analysis and the identification of co-citation bursts. The analysis parameters included annual time slicing from 2014 to 2023, with the selection criteria set using a g-index of 15 and a link retaining factor (LRF) of 2.5 [29]. A g-index of 15 signifies that an author has published at least 15 papers that have collectively received a minimum of 225 citations. An LRF of 2.5 indicates that, on average, each citation in the author’s top publications generates or maintains 2.5 additional citations in future works or related studies.

In this study, various software applications were employed to examine different elements of the literature, including temporal and geographical distributions, networks of author collaborations, and keyword relationships. These tools generate visual outputs, such as knowledge maps, keyword cluster diagrams, and graphical representations, facilitating a comprehensive analysis and interpretation of patterns and significant aspects within the field of study. This study aims to identify emerging research trends and provide valuable insights for academics working in this domain.

## 3. Results

### 3.1. Global publication and citation trends

This study analyzes MSCs-derived exosomes’ development trends and research impact. We analyzed literature from the Web of Science database, covering 2014 to 2023. The data were filtered by language and document type, including 948 publications from 55 countries, 1098 institutions, and 4707 authors. This study’s findings reveal the global development trends and academic impact of research on MSCs-derived exosomes (Fig. 1).

**Fig. 2:**
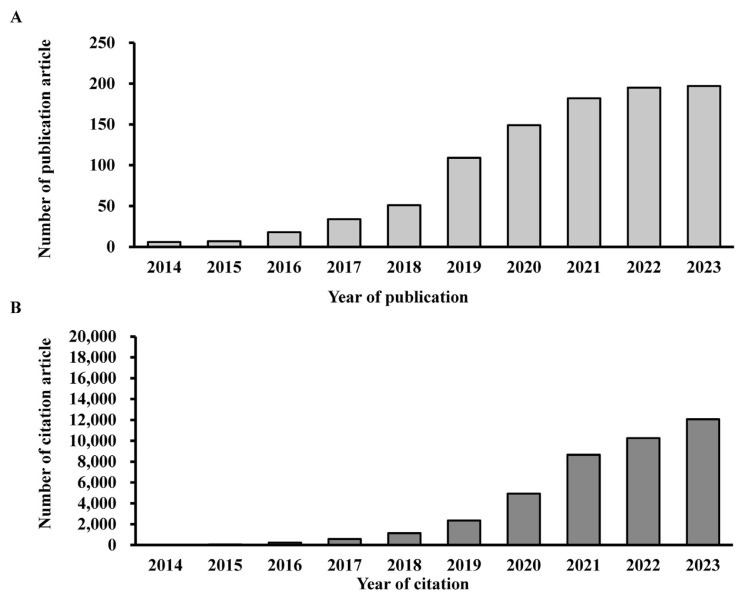
Annual trend in publications and citations on MSCs and exosome research. (A) The growth in published articles from 2014 to 2023, with a significant uptick observed starting in 2018 and reaching its highest point during the 2021–2023 period; (B) The associated rise in citations demonstrates a notable uptick in 2021, 2022, and 2023, suggesting an expanding influence and interest in the research domain focusing on MSCs and exosomes.

As illustrated in Fig. 2A, research on MSCs-derived exosomes has experienced a substantial increase in global publications from 2014 to 2023. The field’s inception in 2014 saw six publications, followed by modest growth, with seven publications in 2015 and 18 in 2016. A significant expansion began in 2017, yielding 34 publications, which increased to 51 in 2018 the year 2019 marked a pivotal moment, with 109 publications indicating rapid progress in the field. Subsequently, annual publication counts have consistently exceeded 100, with 149 in 2020, 182 in 2021, 195 in 2022, and a maximum of 197 in 2023. These findings highlight the growing prominence of MSCs-derived exosomes as an emerging research focus, continually drawing worldwide interest and resources. The increasing academic significance of research on MSCs-derived exosomes is illustrated in Fig. 2B, which shows a steady rise in citation counts. Starting with just ten citations in 2014, the field experienced rapid growth, reaching 55 citations in 2015 and 235 citations in 2016. This upward trend continued, with citations soaring to 575 in 2017 and consistently surpassing 1000 per year from 2018 onward. 2352 citations in 2019 and a significant increase to 4935 in 2020 demonstrated the field’s importance. An important milestone was reached in 2022, when citations exceeded 10,000, culminating in 12,067 citations by 2023. These statistics indicate a swift increase in scholarly and industrial interests in this area. The continuous increase in citations emphasizes the considerable reference value of this body of literature and highlights its crucial role in driving progress in both academic and industrial research contexts. These patterns show that exosomes derived from stem cells are increasingly important in current scientific studies. They show that their influence and importance are growing worldwide.

### 3.2. Global research contributions

**Fig. 3:**
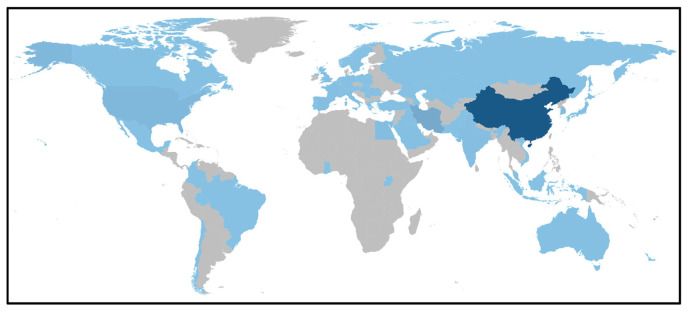
Global distribution of publications on MSCs and exosome research. The global map illustrates the spatial distribution of research publications on MSCs and exosomes. Regions depicted in the darkest shades, such as China, indicate the highest concentration of published studies. Countries in lighter hues represent areas with moderate research output in this field. Gray areas denote regions with minimal or no published research on the topic.

**Fig. 4:**
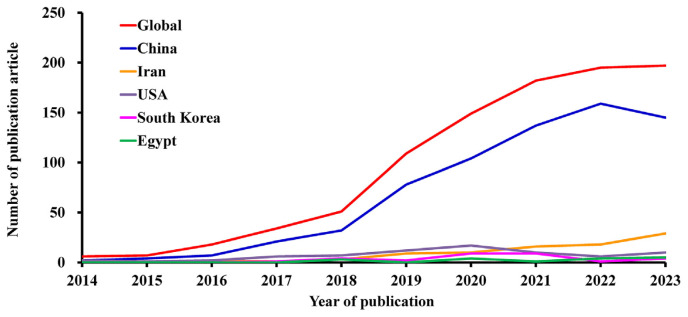
Publication trends in MSCs and exosome research in leading countries. The diagram illustrates publication trends, contrasting global patterns with major contributing nations, including China, Iran, the United States, South Korea, and Egypt. The international publication tally, represented by the red line, shows a dramatic upswing beginning in 2018, reaching its peak around 2021. The blue line represents China, which leads to publication volume showing a noticeable increase from 2018 and maintaining strong output through 2023. Other countries, such as Iran (orange line), the United States (purple line), and South Korea (pink line), have displayed modest but consistent contributions, with slight upward trajectories in recent years.

The study of MSCs-derived exosomes has experienced significant growth since 2014, with research conducted in 55 countries worldwide indicating its international significance and scientific relevance. A geographical distribution map created using AI-Bibliometrix and shown in Fig. 3 reveals China’s dominance in this research area, with the highest number of publications. While Iran and the United States follow China regarding research output, the volume of publications from China significantly surpasses that of both countries combined. This position highlights China’s considerable research investment and contributions to MSCs-derived exosomes. As shown in Table 1, China leads the world with 689 publications, significantly surpassing Iran’s 88 and the United States’ 73 publications. Asian nations comprise six top ten countries, including China, Iran, South Korea, India, Taiwan, and Japan. This predominance highlights the substantial research efforts in Asia, especially in East Asian countries. This trend indicates increasing attention and advancements in MSCs-derived exosome research in these regions. Although China produces the highest number of publications, Japan excels in the quality and influence of its research outputs. Japan is in the lead, with an impressive average of 113.72 citations per publication. The United States was second with 84.97 citations, and Taiwan was third with 53.84 citations. This underscores Japan’s prominent role in generating highly influential research despite publishing fewer papers than China. The global publication patterns in research on MSCs-derived exosomes are illustrated in Fig. 4, focusing on China’s leading position. The graph reveals a steep increase in China’s publication output, signifying its increasing prominence in the study area. Furthermore, Iran has shown a consistent upward trend in the number of publications, suggesting its ongoing commitment to research on MSCs-derived exosomes. These results highlight the research landscape for MSCs-derived exosome studies. They show how scientific work has spread worldwide and which countries are leading the way in making progress in this area. China is the leading contributor, with substantial research output from other Asian countries and the United States.

### 3.3. International collaboration patterns

**Fig. 5:**
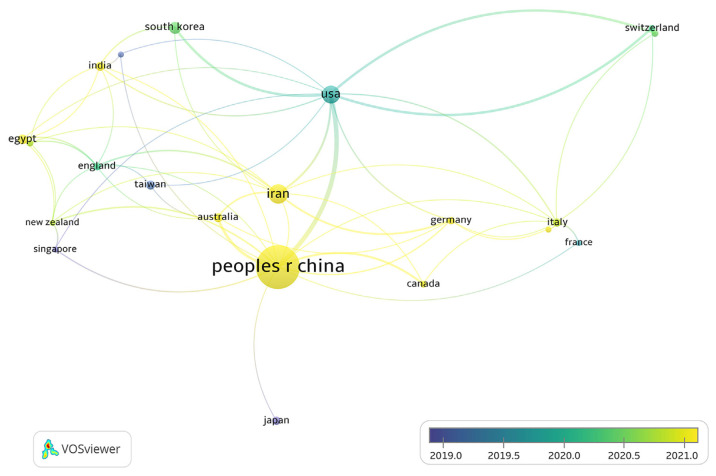
International collaboration network on MSCs and exosome research. The VOSviewer network visualization illustrates the collaborative connections among nations in MSCs and exosome studies. The largest node represents China, highlighting its significant partnerships with Iran, the United States, and Italy. Line thickness indicates the strength of cooperation, while the color gradient from blue to yellow reflects increasing collaboration intensity from 2019 to 2021.

**Fig. 6:**
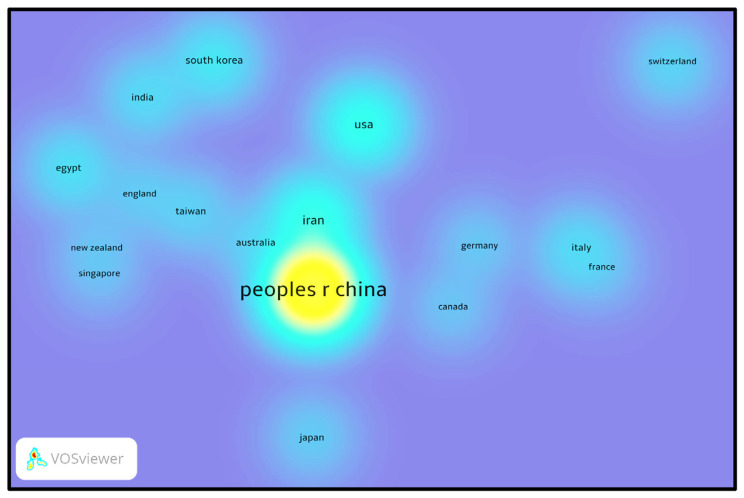
Heatmap of international research activities on MSCs and exosomes. The image illustrates a heatmap illustrating the concentration of scientific output in MSCs and exosome studies globally. Warmer hues, such as yellow, indicate higher productivity, with China leading the field. Countries like the United States, Iran, and Germany demonstrate moderate research activity, while cooler blue regions represent areas with lower output.

Figs. 5 and 6 were created using VOSviewer software. They show a complete picture of the global networks of countries that have worked together to produce at least five publications on MSCs-derived exosomes. Fig. 5 depicts the co-authorship connections between nations, where the size of each circle corresponds to the number of publications produced by that country. Larger circles represent nations with higher publication output. Lines connecting the circles signify co-authorship relationships between countries, with thickness indicating the frequency of collaborations. The largest circle, representing the highest number of publications, suggests that China is the industry leader in this field. The thickest connecting line shows the most significant collaboration between China and the United States. While ranking second in publication output, Iran has minimal collaborative ties with China, suggesting that Iranian research is conducted independently or with a regional focus. Purple-colored nations, such as Taiwan, Japan, and Singapore, are considered pioneers in MSCs-derived exosome research, signifying their early involvement in this field. Conversely, countries highlighted in yellow, such as China, are considered latecomers in this study area. Interestingly, despite its delayed entry, China swiftly became a frontrunner in research output. This rapid advancement shows China’s capacity to quickly increase its scientific contributions and hints at its potential to become a future leader in this domain.

The heatmap in Fig. 6 displays the concentration of national collaborative networks. Intense colors indicate higher levels of co-authorship and joint publication activities, whereas muted shades represent lower levels of collaboration. China has emerged as the focal point, exhibiting an exceptionally high concentration of publication data points, emphasizing its leading position in research output and scholarly contributions. This concentrated cluster of data further highlights China’s widespread participation in global collaboration and the expansive nature of its research connections. Figs. 5 and 6 collectively show China’s swift ascent to a leading position in research on exosomes derived from MSCs. This is evident not only in terms of research volume but also in international collaborative efforts, especially in the United States. Nevertheless, other nations, such as Japan, Taiwan, and Iran, also make notable contributions, enriching the global research landscape in various ways.

These collaboration patterns underscore the crucial role of cross-border scientific cooperation in advancing the emerging and influential field of study.

### 3.4. Institutional leadership and collaboration

According to Table 2, Chinese academic institutions are at the forefront of research on MSCs-derived exosomes, with universities from China occupying leading positions in terms of both publication quantity and citation influence. The top-ranking institution, Shanghai Jiao Tong University, has produced 37 publications and garnered 3651 citations, yielding a remarkable average of 98.67 citations per article. Nanjing Medical University closely followed with 35 publications, while Shandong University secured the third position with 30 publications. These institutions’ significant average citation counts reflect the widespread acclaim and influence of their scientific contributions while also demonstrating high research productivity and exceptional quality. Two Iranian medical universities, Shahid Beheshti and Tehran, hold prominent positions in the global research arena, ranking fifth and seventh, respectively. Their presence among the top ten institutions highlights Iran’s academic prowess in this field, mainly through producing influential, high-quality research publications with significant worldwide impact. When evaluating the research impact, the average number of citations per publication is a crucial indicator. Among the institutions studied Shanghai Jiao Tong University has an impressive 98.67 citations per paper. Jiangsu University and Zhejiang University also demonstrated notable performance, averaging 71.08 and 67.83 citations, respectively. In Taiwan, several institutions have contributed significantly to MSCs-derived exosome research. China Medical University, Asia University, and Kaohsiung Chang Gung Memorial Hospital have garnered considerable academic recognition with average citation counts of 68.67, 82.33, and 101.67, respectively. These figures highlight Taiwan’s important role in advancing this area of study.

**Fig. 7:**
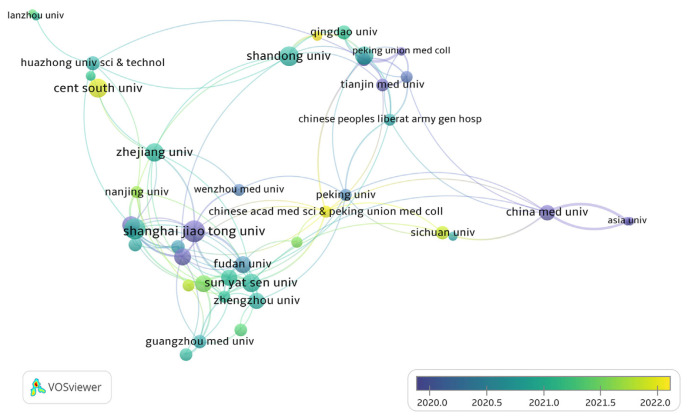
Institutional collaboration network in MSCs and exosome research. The VOSviewer image illustrates the collaborative relationships between Chinese academic and research organizations involved in studies on MSCs and exosomes. Key institutions, including Shanghai Jiao Tong University, Zhejiang University, and Peking Union Medical College, hold prominent positions within the network with solid partnerships indicated by inter-connecting lines. The color spectrum from blue to yellow represents the timeline of collaborations, with recent partnerships shown in yellow.

**Fig. 8:**
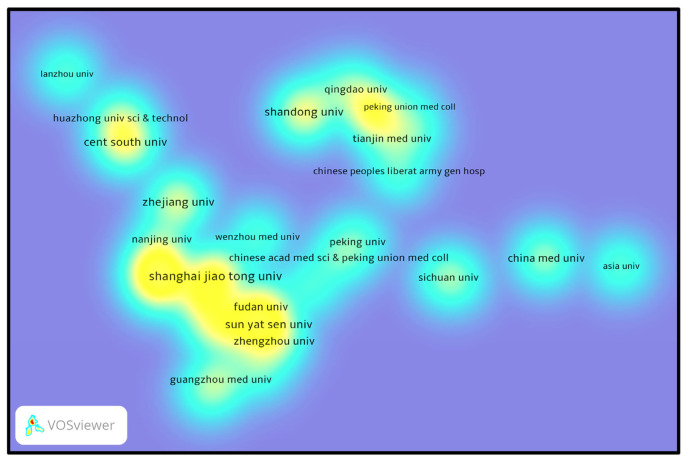
Heatmap of institutional research activities on MSCs and exosome research. The image displays a heatmap illustrating the concentration of research efforts on MSCs and exosomes across various Chinese academic institutions. The brighter yellow areas signify universities with greater research output, with Shanghai Jiao Tong University, Zhejiang University, and Peking Union Medical College emerging as prominent contributors. Cooler blue tones indicate institutions with lower activity.

The collaboration networks among institutions with at least six publications are depicted in Figs. 7 and 8, using VOSviewer’s co-authorship analysis. In Fig. 7, the size of each circle represents the number of publications, whereas the connecting lines indicate co-authorship connections. Thick lines represent more vital collaboration.

The giant circles belong to Shanghai Jiao Tong University, Zhejiang University, and Shandong University, signifying their substantial publication output and prominent role in this field. The thick line connecting Shanghai Jiao Tong University and Zhejiang University provides evidence of their collaborative solid ties, also present in these institutions. The chronological analysis depicted in Fig. 7 demonstrates a notable uptick in scientific endeavors at institutions such as Shanghai Jiao Tong University and Nanjing Medical University post-2020, indicating their growing prominence and scholarly productivity during this timeframe. Taiwan-based China Medical University (CMU) and Asia University stand out as early pioneers in MSCs-derived exosome studies, with their research activities centered around the year 2020 on average. The abundance of yellow tones in the chronology, indicating more contemporary studies, suggests a general trend of increased research productivity and collaboration among institutions starting in 2020. Greater global participation and resource sharing have been driving forces behind this trend.

The heatmap in Fig. 8 illustrates the concentration of collaborative networks among institutions. Areas with higher brightness indicate more excellent collaborative activity. In this field, Shanghai Jiao Tong University, Zhejiang University, and Shandong University have emerged as central figures, demonstrating frequent research engagement and notable scholarly impact. The intense bright areas connecting Shanghai Jiao Tong University and Zhejiang University suggest robust collaborative relationships, underscoring their prominent roles. These cooperative efforts have made significant contributions to the research environment, which shows how important they are to advancing the field. In conclusion, Chinese academic institutions, with Shanghai Jiao Tong University, Nanjing Medical University, and Zhejiang University at the forefront, are pivotal in producing research and fostering collaboration in MSCs-derived exosomes. Additionally, Taiwan and Iran made notable contributions, particularly in terms of the impact of their research on citations. This reflects a wide-ranging, internationally connected research endeavor that has seen continued expansion, particularly in the years following 2020.

### 3.5. Journal rankings and impact in MSCs-derived exosome

The 2022 Journal Citation Reports (JCR) provides impact factors (IF) for journals analyzed in Table 3. This assessment showcases the most influential journals publishing studies on MSCs-derived exosomes, indicating their scholarly impact and reputation in the broader scientific field. At the forefront of stem cell research publications is “Stem Cell Research & Therapy,” which boasts 63 articles and a notable impact factor of 7.5, placing it in the prestigious Q1 quartile. The number of its published works and the regularity with which academics refer to its research serve as evidence for its remarkable standing. This journal’s widespread influence further solidifies its status as a premier outlet for cutting-edge research in stem cell science. Ranking second is “Stem Cells International,” which has published 25 articles and has an impact factor of 4.3, positioning it in the Q3 quartile. Although it has produced fewer publications, this journal maintains significant academic relevance, albeit with less influence than the Q1 journals. Its inclusion among the top three demonstrates the continued relevance and value of its contribution to stem cell research. In third place, “Frontiers in Cell and Developmental Biology” has published 18 articles and has an impact factor of 5.5, securing its place in the Q2 quartile. The journal’s impressive citation metrics indicate its significant impact on cell and developmental biology.

Despite having a lower impact factor than Stem Cell Research & Therapy,” it continues to play a crucial role in disseminating vital research findings in related fields.

Several other journals have exerted considerable academic influence. For example, “Life Sciences” is a highly respected publication with an impact factor of 6.1 and a Q1 quartile ranking. Furthermore, the “International Journal of Molecular Sciences” boasts an impact factor of 5.6 and holds a Q2 quartile position, demonstrating its importance and credibility in molecular sciences and its substantial effect on associated stem cell studies. Conversely, the “Journal of Biomaterials and Tissue Engineering” showed a significantly lower impact factor of 0.1, thus positioning it in the Q4 quartile. The journal appears to have few citations and little impact on academic circles, indicating that experts in the field do not frequently cite published research. Examining these impact factors provides researchers with crucial insights. Scholarly publications with elevated impact factors, particularly those ranked in the top quartile (Q1), are typically held in higher esteem within academic circles and tend to feature research that garners more citations. Consequently, these journals are often the preferred choice for scholars to enhance the reach and impact of their studies. The relative positioning of journals, as seen in their impact factor and quartile classification, also sheds light on the significance of publications within particular research fields. This research provides a comprehensive overview of the academic landscape of MSCs-derived exosome studies, enabling scientists to make well-informed choices about manuscript submission. Additionally, it highlights the key journals driving conversation and progress in this rapidly evolving study area.

### 3.6. Journal influence and collaboration in stem cell research

**Fig. 9:**
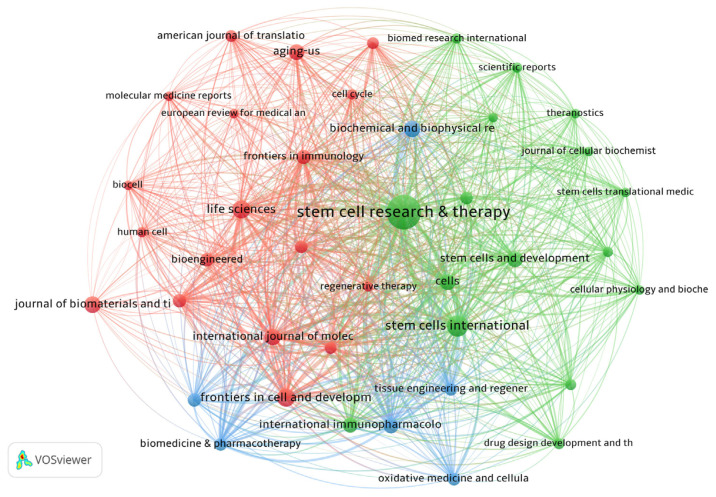
Co-citation network of journals on MSCs and exosome research. The VOSviewer network visualization illustrates the co-citation patterns among journals in the MSCs and exosome research domains. The size of each node corresponds to the journal’s citation count, whereas the connecting lines indicate the strength of the co-citation relationships. Prominent journals, such as Stem Cell Research & Therapy, Stem Cells International, and Life Sciences, have emerged as pivotal nodes in the network, highlighting their substantial impact in this research area. The visualization uses various colors to denote clusters of journals that frequently reference one another.

The VOSviewer was used to create a bibliographic coupling analysis, as shown in Fig. 9. This visualization depicts the interconnections among journals in the MSCs-derived exosome field based on shared keywords in publications containing five or more articles. The resulting network illustrates the academic relationships and influences between these journals, highlighting citation patterns and common terminology usage within the research area. The network map reveals “Stem Cell Research & Therapy” as a pivotal hub, demonstrating its significant impact. Its prominent position highlights robust bibliographic coupling links, implying that this publication generates substantial influential research and engages in frequent citation exchanges with other journals. This emphasizes its crucial function as a primary outlet for circulating stem cell research findings, promoting the exchange of knowledge and propelling academic advancements in this domain. Likewise, “Stem Cells International” and “Life Sciences” emerged as prominent nodes, exhibiting robust links to numerous other journals. These interconnections underscore their academic importance and dynamic involvement in stem cell research. Their strong citation networks and collaboration with other publications suggest that these journals influence research directions and contribute significantly to global scientific dialogue in associated disciplines. As illustrated in the map, the intricate web of citations and collaborations among prominent journals establishes a solid scholarly ecosystem. This interconnectivity promotes the circulation and spread of information, enabling discoveries published in one journal to shape and enhance research in other journals. The tight bibliographic links between these publications underscore their combined influence on the scientific community’s understanding of stem cell-derived exosomes and their associated fields. In conclusion, the analysis of bibliographic coupling demonstrates the interconnected nature of prominent journals such as “Stem Cell Research & Therapy,” “Stem Cells International,” and “Life Sciences” within a closely linked scholarly community. These publications play a dual role by functioning as key information sources and fostering the field’s growth through citation-based collaboration, ultimately strengthening the collective knowledge foundation of the academic sphere.

### 3.7. Journal influence and research activity in stem cell research

**Fig. 10:**
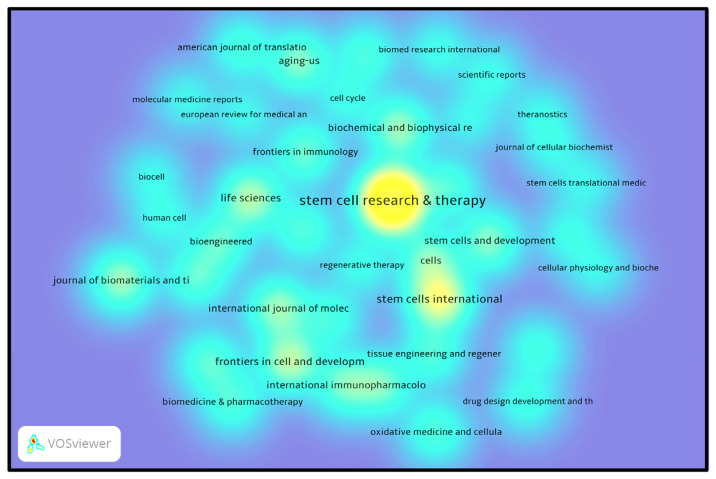
Heatmap of journal co-citation intensity in MSCs and exosome research. The image displays a heatmap illustrating the strength of journal co-citation connections in MSCS and exosome studies. Warmer hues (yellow) represent journals with higher co-citation frequencies, with Stem Cell Research & Therapy, Stem Cells International, and Life Sciences exhibiting the most intense relationships. Cooler blue shades signify lower co-citation levels.

Fig. 10 shows a heatmap of the journals that illustrates the concentrated areas of research activity and scholarly impact within various publications in the stem cell research domain. This visualization identifies crucial journals that significantly contribute to advancing knowledge in the field. The image features “Stem Cell Research & Therapy” as the most notable node, reflecting its high occurrence frequency and number of citations. This journal is a central hub for academic activity, demonstrating its substantial influence and significance within the scientific community. Its position as the most intense region on the heatmap underscores its considerable attention and crucial role in shaping discussions in stem cell research. Other scientific journals, such as “Stem Cells International,” “Cells,” and “Life Sciences,” Life Sciences, have also exhibited considerable research influence. These publications are depicted as bright areas on the heatmap, indicating substantial research output and extensive citations in academic circles. Their inclusion emphasizes their importance for spreading critical studies and helps to keep learning more about MSCs-derived exosomes and related topics. Finally, the heatmap analysis in Fig. 10 and previous studies of journal publication output, impact factors, and bibliographic coupling associations provide a complete picture of the most crucial stem cell research journals. This investigation underscores the scholarly prominence of major publications such as “Stem Cell Research & Therapy” and reveals the intricate network of collaboration and reciprocal citations among critical journals.

These results provide crucial information for scientists seeking to publish in journals with substantial research activity and influence while emphasizing the interconnected nature of academic endeavors in this dynamic and rapidly progressing field.

### 3.8. Keyword analysis

We used VOSviewer and CiteSpace to perform a complete analysis of the keyword co-occurrence networks and clustering. This reveals the main research themes and patterns in MSCs-derived exosome studies. Furthermore, examining the top 20 keywords exhibiting the most significant citation bursts provides insight into increased scholarly interest in specific areas between 2014 and 2023.

**Fig. 11:**
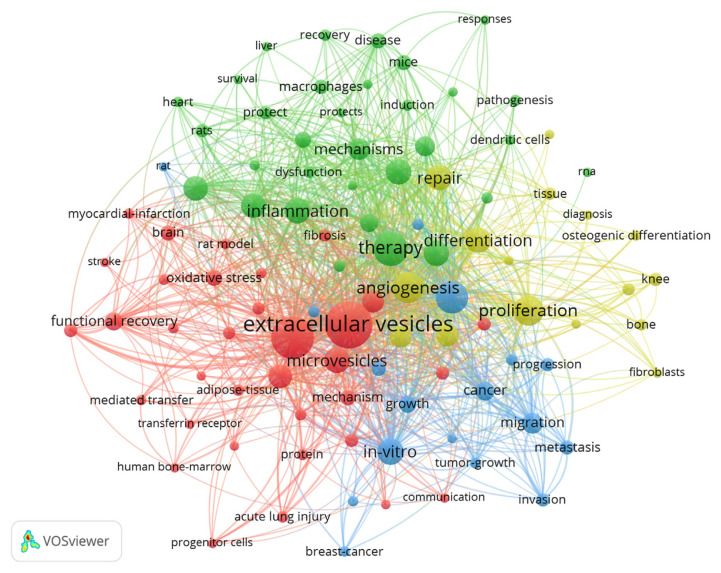
Keyword co-occurrence network in MSCs and exosome research. The VOSviewer network visualization illustrates the co-occurrence of keywords in research related to MSCs and exosomes. Node size correlates with keyword frequency, whereas inter-node connections represent the co-occurrence of keywords in the studies. The network’s core consists of prominent terms, such as extracellular vesicles, inflammation, proliferation, angiogenesis, and repair, highlighting their significance in this field of study. Distinct color-coded clusters represent thematically linked groups of frequently co-occurring keywords.

The keyword co-occurrence analysis presented in Fig. 11 identifies four primary research clusters, each representing a distinct area of focus within the field. Cluster 1 (Red): Tissue Regeneration and Functional Restoration. This cluster consisted of 34 keywords associated with using MSCs-derived exosomes to enhance tissue regeneration and restore functionality. Key phrases such as “acute lung injury,” “bone marrow,” “functional recovery,” “ischemia-reperfusion injury,” “myocardial infarction,” and “stroke” show that exosomes may be able to help with a wide range of short- and long-term health problems. Research in this cluster focuses on harnessing exosomes to repair damaged tissues, with a particular emphasis on their therapeutic applications in cardiovascular and neurological disorders, among other areas.

Cluster 2 (Green): Immune Regulation and Inflammation Management. This cluster comprised 32 keywords that emphasized the role of MSCs-derived exosomes in regulating immune responses and managing inflammation. It includes crucial terms such as “activation,” “apoptosis,” “inflammation,” “macrophages,” “pathway,” and “therapy.” The focus of this cluster suggests that these exosomes show significant promise for treating inflammatory disorders by targeting the immune system mechanisms. This indicates that exosomes from MSCs play a vital role in modulating immune function and controlling inflammation. They are promising candidates for developing therapies aimed at autoimmune and inflammatory conditions.

Cluster 3 (Yellow): Bone Tissue Restoration and Regeneration. This cluster encompasses 16 keywords centered on bone tissue restoration and regeneration and highlights the regenerative potential of MSCsl-derived exosomes. Key terms such as “angiogenesis,” “bone,” “differentiation,” “regeneration,” “repair,” and “tissue” emphasize the role of exosomes in mending bone and cartilage structures. This cluster underscores the potential application of exosomes in orthopedic treatments, particularly for promoting healing in cases involving bone damage or loss.

The fourth cluster, Cluster 4 (Blue), consists of 17 keywords focused on applying MSCs-derived exosomes in cancer research. This group includes significant terms such as “biomarkers,” “breast cancer,” “drug delivery,” “metastasis,” and “tumor growth.” This emphasizes the increasing importance of exosomes in cancer detection and treatment, particularly their potential as drug carriers and indicators of cancer presence. These findings suggest that exosomes may be crucial in enhancing cancer therapy and facilitating targeted anticancer drug administration.

**Fig. 12:**
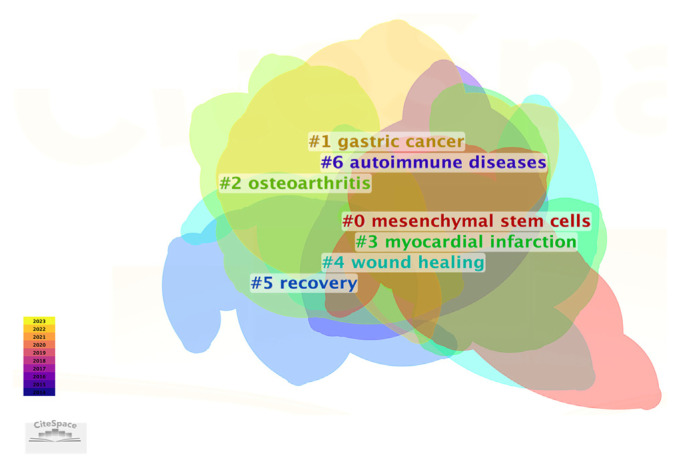
Thematic clusters in MSCs and exosome research. CiteSpace visualization illustrates the primary research themes in MSCs and exosomes over time, represented by intersecting regions. Significant research areas have been identified, including MSCs, gastric cancer, osteoarthritis, myocardial infarction, wound healing, recovery, and autoimmune diseases. The color gradient, ranging from purple (2016) to yellow (2023), reflects the chronological progression of research focus.

An extensive examination of the 15 most significant keywords exhibiting intense citation bursts is presented in Fig. 12, illustrating the periods of heightened scholarly interest from 2014 to 2023. This visualization aids in identifying the changing focal points within the field, providing a chronological overview of when specific research themes gained traction. Keywords experiencing citation bursts indicate shifts in research priorities and underscore emerging areas of interest. For example, exosome-based therapies targeting inflammation and cancer have garnered considerable attention in recent years, reflecting the evolving research landscape in this domain. This keyword analysis revealed the dominant themes and research directions in MSCs-derived exosome studies over the past decade. These four clusters, focusing on tissue repair, immune response, bone regeneration, and cancer, highlight the multifaceted potential of exosomes in therapeutic applications. The immune regulation cluster shows how MSC-derived exosomes may be able to change immune responses, which is becoming increasingly important for treating autoimmune and inflammatory diseases.” In the future, researchers will probably look into how exosomes specifically change the functions of immune cells, such as how macrophages polarize and how lymphocytes work, to create targeted treatments for immune-related diseases. Tissue regeneration clusters underscore the essential role of exosomes in facilitating tissue repair, particularly in cardiovascular diseases, neural injuries, and bone regeneration. As the demand for regenerative medicine increases, future studies should explore how exosomes enhance tissue healing, including angiogenesis and anti-inflammatory effects. The research will also aim to improve exosome delivery systems for more precise and practical therapeutic applications.

Citation burst analysis further illustrates the dynamic nature of the field, providing valuable insights into periods of intense focus on particular topics. Together, these findings offer researchers a clear view of major research trends, enabling a deeper understanding of where the field is heading and identifying critical areas for future exploration.

### 3.9. Burst analysis of keyword trends

**Fig. 13:**
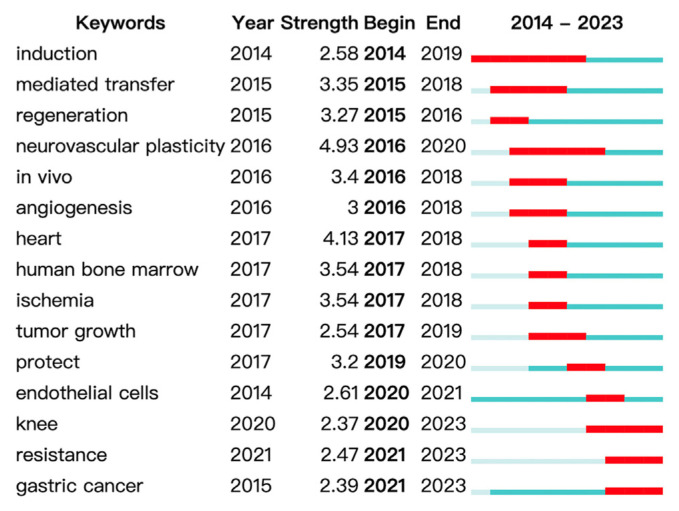
Emerging keywords in MSCs and exosome research. This figure presents emerging keywords, their burst strength (indicating growing importance), the start year of the trend, and its duration (shown by red bars). Topics such as neurovascular plasticity (2016–2020), angiogenesis (2016–2018), and heart research (2017–2018) have gained attention over time. Recent trends, including “resistance” (2021–2023) and “gastric cancer” (2021–2023), suggest he ongoing expansion of research in these fields.

The burst analysis of the keywords depicted in Fig. 13 illuminates the potential and future trajectories for medical applications of exosomes derived from stem cells.

This examination reveals three primary areas of expansion and an emerging emphasis.

1. Cardiovascular Diseases: The analysis of bursts revealed a persistent and robust interest in utilizing exosomes derived from MSCs for treating cardiovascular ailments. The terms associated with “myocardial infarction,” “ischemia-reperfusion injury,” and “acute lung injury” acute lung injury demonstrate an increasing acknowledgment of exosomes as potential therapeutic tools for repairing heart and blood vessel damage. This emphasis indicates that future studies will likely continue to investigate and optimize the application of exosomes to improve tissue regeneration and functional recovery among individuals with cardiovascular disorders.
2. Cancer Treatment: The surge in cancer-related terms like “biomarkers”, “drug delivery” and “metastasis” highlights the growing importance of exosomes in cancer research. These vesicles are increasingly recognized as crucial tools for detecting cancer, delivering targeted treatments, and impeding tumor growth. Analysis of burst patterns suggests that current and forthcoming research will likely focus on creating exosome-based therapies, with particular emphasis on improving the effectiveness and accuracy of cancer treatments.
3. Regeneration of Bone Tissue: Burst analysis also highlighted the significance of bone tissue regeneration. The presence of keywords associated with “bone,” “regeneration,” and “repair” emphasizes the promising applications of exosomes derived from mesenchymal to facilitate the healing and restoration of bone and cartilage tissues. This indicates that future studies should explore their potential in the fields of orthopedics and regenerative medicine, focusing on innovative treatments for bone-related injuries and degenerative disorders.

The analysis of bursts depicted in Fig. 13 offers crucial insights into the future trajectory of research on MSCs-derived exosomes. By emphasizing on cardiovascular disorders, cancer therapies, and bone tissue restoration, these results underscore the domains in which exosomes are expected to yield substantial clinical benefits. This examination not only apprises scientists of current research trends, but also presents a strategic plan for future investigations, propelling the progress of exosomes in medical treatments and therapeutic applications.

## 4. Discussion

Exosomes made from MSCs have been the subject of extensive research thanks to growing awareness of their potential uses in regenerative medicine. Our AI-bibliometric study, which examines worldwide publications from 2014 to 2023, reveals an exponential rise in publications and their citation impact, particularly since 2014. This trend indicates heightened interest in MSCs-derived exosomes [30–33]. These exosomes facilitate intercellular communication and transport bioactive molecules, promote tissue regeneration, and alleviate disease. This change means exosome-focused therapies will replace conventional cell-based ones [30,33]. This makes exosomes an exciting area for future therapeutic interventions.

China has a high publication output, frequent citations, and significant studies, which show that it is a leader in MSCs-derived exosome research. Contributing factors include substantial government funding, national policies that strategically support biotechnology and regenerative medicine, and the establishment of specialized research centers in China. These elements have been crucial in the rapid advancement and significant presence of Chinese institutions in this field. The United States continues to contribute substantially, producing high-quality research that underscores the field’s competitive yet cooperative global nature. Collaboration between nations, especially China and the U.S., plays a crucial role in fostering innovation and addressing the challenges associated with clinical translation. These challenges include improving exosome production methods, establishing standardized therapeutic protocols, and gaining a more comprehensive understanding of the underlying mechanisms of action [32].

The AI-bibliometric study also highlights a range of research areas within this domain. The main ideas include how exosomes made from MSCs can heal damaged tissues, change immune responses, and possibly even treat cancer and grow new bones. These results illustrate the adaptability of exosomes in addressing various acute and chronic medical conditions. This analysis substantiates the pivotal role of MSCs-derived exosomes in advancing regenerative medicine. The field has experienced significant growth, evolving from fundamental areas such as tissue regeneration to more specialized applications, including cancer treatment, therapies for neurodegenerative disorders, and recovery from myocardial infarction. The increasing research on exosomes’ immune-modulatory capabilities suggests their potential in addressing inflammation-related conditions, further extending their therapeutic applications. Extensive international cooperation, particularly between leading countries like China and the United States, has been crucial to the rapid development of this field. The main advantage of this study lies in its thorough examination of research trends in MSCS-derived exosomes and their global influence. Utilizing bibliometric tools, we have identified vital contributors, emerging patterns, and research clusters that shape current and future investigations. Furthermore, the study emphasizes the importance of global collaboration, which has been instrumental in driving research progress and fostering innovation. However, the research has certain limitations. Primarily, it relied heavily on the Web of Science database, which may have excluded relevant studies from other repositories such as Scopus or PubMed. Additionally, language and document type filters may have omitted some critical research, resulting in a less comprehensive analysis. Moreover, the study did not extensively explore ongoing clinical trials, essential for understanding exosome-based treatments’ clinical application and effectiveness.

To advance the field, several vital areas require attention. For example, more research is needed to fully understand how exosomes made from MSCs work, such as how they move around the body, interact with target cells, and their pharmacokinetic properties [16]. Gaining a deeper understanding of these processes is vital for enhancing therapeutic effectiveness and ensuring precise delivery. Another significant challenge is scalability, as current exosome production techniques are not yet suitable for large-scale clinical use [31]. Developing efficient, scalable production methods is essential to ensuring consistent quality in exosome-based therapeutic approaches.

Looking ahead, MSCs-derived exosomes are expected to impact personalized healthcare critically. Scientists can customize treatments for specific patients by modifying the contents of exosomes, such as incorporating particular proteins, mRNAs, or microRNAs. This approach offers targeted therapies for complex medical conditions [31]. Additionally, the advancement of this field would be significantly enhanced by conducting more comprehensive clinical studies and trials, especially in areas like cancer immunotherapy and regenerative medicine. These investigations would help evaluate the safety, effectiveness, and long-term outcomes of therapies based on exosomes.

Despite substantial progress in MSCs-derived exosomes, significant obstacles and knowledge gaps must be addressed. Scientists must keep researching exosomes to fully utilize their healing potential in regenerative medicine and other areas. This will be easier if scientists work together worldwide and improve manufacturing processes.

## 5. Conclusion

Prominent scientific institutions and nations worldwide are driving the expansion of MSCs-derived exosome research. This study’s AI-bibliometric analysis highlights the importance of cross-border cooperation and multidisciplinary strategies in advancing the field. As research progresses, MSCs-derived exosomes are expected to play an increasingly significant role in regenerative medicine and cell-based therapies, potentially offering novel treatments for various medical conditions. However, further studies are needed to harness their therapeutic capabilities fully. Critical areas for investigation include understanding their modes of action, enhancing targeted delivery techniques, and addressing challenges related to large-scale production and clinical implementation. Ongoing research efforts suggest that MSCs-derived exosomes could revolutionize future medical treatments.

## 6. Limitations

While this research offers a comprehensive analysis of global research trends using AI-bibliometric approaches, it has limitations. One notable constraint is the study’s reliance solely on data from the Web of Science database, which may have omitted relevant research from other sources. Furthermore, applying language and document-type filters could have led to the omission of significant studies from the analysis.

## Supplementary Information









## Figures and Tables

**Table 1:** Countries with the most publications in MSCs-derived exosome research.

NO	Country	Documents	Citation	Average citation
1	CHINA	689	28361	41.16
2	IRAN	88	2929	33.28
3	USA	73	6203	84.97
4	SOUTH KOREA	30	1437	47.90
5	EGYPT	17	368	21.65
6	INDIA	15	385	25.67
7	AUSTRALIA	13	282	21.69
8	TAIWAN	13	700	53.85
9	ITALY	12	406	33.83
10	JAPAN	11	1251	113.73
11	ENGLAND	10	400	40.00
12	SPAIN	10	876	87.60
13	GERMANY	9	338	37.56
14	CANADA	8	298	37.25
15	SWITZERLAND	8	742	92.75
16	SAUDI ARABIA	7	148	21.14
17	SERBIA	7	571	81.57
18	FRANCE	6	819	136.50
19	ISRAEL	6	516	86.00
20	NEW ZEALAND	6	226	37.67
21	TURKEY	6	152	25.33
22	SINGAPORE	5	967	193.40
23	CHILE	4	590	147.50
24	IRAQ	4	103	25.75
25	TURKIYE	4	7	1.75
26	MALAYSIA	3	90	30.00
27	NETHERLANDS	3	127	42.33
28	PAKISTAN	3	8	2.67
29	VIETNAM	3	83	27.67
30	AZERBAIJAN	2	7	3.50
31	GHANA	2	17	8.50
32	GREECE	2	11	5.50
33	INDONESIA	2	83	41.50
34	LEBANON	2	60	30.00
35	ROMANIA	2	6	3.00
36	RUSSIA	2	137	68.50
37	SLOVAKIA	2	123	61.50
38	AUSTRIA	1	132	132.00
39	BELGIUM	1	277	277.00
40	BRAZIL	1	50	50.00
41	COLOMBIA	1	89	89.00
42	CYPRUS	1	42	42.00
43	HUNGARY	1	38	38.00
44	KAZAKHSTAN	1	3	3.00
45	KUWAIT	1	3	3.00
46	MEXICO	1	121	121.00
47	NORWAY	1	14	14.00
48	PALESTINE	1	17	17.00
49	POLAND	1	149	149.00
50	PORTUGAL	1	94	94.00

**Table 2:** The top 10 research institutions working on MSCs-derived exosomes.

No	Affiliations	Documents	Citations	Average citations
1	Shanghai jiao tong univ	37	3651	98.67
2	Nanjing med univ	35	1724	49.26
3	Shandong univ	30	1089	36.30
4	Cent south univ	29	612	21.10
5	Sun yat sen univ	27	1229	45.52
6	Capital med univ	26	755	29.04
7	Zhejiang univ	26	1752	67.38
8	Jiangsu univ	25	1777	71.08
9	Fudan univ	23	1284	55.83
10	Southern med univ	23	826	35.91

**Table 3:** Top 10 journals in MSCs research.

No	Journal	Documents	Citations	Average citations
1	Stem cell research & therapy	63	5353	84.97
2	Stem cells international	25	1227	49.08
3	Frontiers in cell and developmental biology	18	306	17.00
4	Life sciences	17	758	44.59
5	Biochemical and biophysical research communications	15	784	52.27
6	International journal of molecular sciences	15	1154	76.93
7	Journal of biomaterials and tissue engineering	15	4	0.27
8	Aging-us	13	530	40.77
9	Cells	13	1331	102.38
10	Stem cells and development	13	1022	78.61
