# Supplementary material for: Artificial intelligence (AI)-powered bibliometric analysis of global trends in mesenchymal stem cells (MSCs)-derived exosome research: 2014–2023
Source: Biomedicine (Taipei). 2024 Dec 1;14(4):61–77. doi: 10.37796/2211-8039.1470 (PMC11703396; doi:10.37796/2211-8039.1470)
Supplement: Supplementary file 1 [file bmed-14-04-061-s001.docx]

**October 21, 2024**

Editor-in-Chief
Biomedicine

Dear Editor,

We and our team members would like to express our sincere gratitude for accepting our paper, "**AI-Powered Bibliometric Analysis of Global Trends in MSC-Derived Exosome Research: 2014-2023**," for publication in **Biomedicine (BIOMED_1470)**. In response to the reviewers' feedback, we have carefully implemented all suggested changes and revised the manuscript.

After thorough proofreading to ensure accuracy and readability, we are confident that the revised version is ready for publication. The review process has significantly enhanced the quality of our work, and we greatly appreciate the insight.

We would like to reiterate our thanks for the opportunity to contribute to the field of **Biomedicine**. We eagerly anticipate the impact of our research when featured in the upcoming issue.

Sincerely,

Dr. Jai-Sing Yang

Department of Medical Research

China Medical University Hospital

China Medical University, Taichung, Taiwan

Email: jaisingyang@gmail.com
